# Prediction of New Risk Genes and Potential Drugs for Rheumatoid Arthritis from Multiomics Data

**DOI:** 10.1155/2022/6783659

**Published:** 2022-01-31

**Authors:** Anteneh M. Birga, Liping Ren, Huaichao Luo, Yang Zhang, Jian Huang

**Affiliations:** ^1^School of Life Science and Technology, University of Electronic Science and Technology of China (UESTC), Chengdu, China; ^2^School of Health Care Technology, Chengdu Neusoft University, Chengdu, China; ^3^Department of Clinical Laboratory, Sichuan Cancer Hospital & Institute, Sichuan Cancer Center, University of Electronic Science and Technology of China (UESTC), Chengdu, China; ^4^Innovative Institute of Chinese Medicine and Pharmacy, Chengdu University of Traditional Chinese Medicine, Chengdu, China

## Abstract

Rheumatoid arthritis (RA) is an autoimmune and inflammatory disease for which there is a lack of therapeutic options. Genome-wide association studies (GWASs) have identified over 100 genetic loci associated with RA susceptibility; however, the most causal risk genes (RGs) associated with, and molecular mechanism underlying, RA remain unknown. In this study, we collected 95 RA-associated loci from multiple GWASs and detected 87 candidate high-confidence risk genes (HRGs) from these loci via integrated multiomics data (the genome-scale chromosome conformation capture data, enhancer-promoter linkage data, and gene expression data) using the Bayesian integrative risk gene selector (iRIGS). Analysis of these HRGs indicates that these genes were indeed, markedly associated with different aspects of RA. Among these, 36 and 46 HRGs have been reported to be related to RA and autoimmunity, respectively. Meanwhile, most novel HRGs were also involved in the significantly enriched RA-related biological functions and pathways. Furthermore, drug repositioning prediction of the HRGs revealed three potential targets (ERBB2, IL6ST, and MAPK1) and nine possible drugs for RA treatment, of which two IL-6 receptor antagonists (tocilizumab and sarilumab) have been approved for RA treatment and four drugs (trastuzumab, lapatinib, masoprocol, and arsenic trioxide) have been reported to have a high potential to ameliorate RA. In summary, we believe that this study provides new clues for understanding the pathogenesis of RA and is important for research regarding the mechanisms underlying RA and the development of therapeutics for this condition.

## 1. Introduction

Rheumatoid arthritis (RA) is an autoimmune and inflammatory disease in which the immune system mistakenly attacks healthy joint tissues, thereby causing inflammation that primarily affects the joints [1]. It is a multifactorial disease involving complex traits affected by many genetic and environmental factors, as well as the potential interactions among these factors [2]. Although the etiology underlying RA development is not fully understood, investigators have determined that abnormal immune system responses are the core cause of RA-associated inflammation and joint destruction [3].

Currently, there is no cure for RA. Disease-modifying antirheumatic drugs (DMARDs) still represent the main treatment strategy for RA. These drugs mainly act on the immune system and slow the progression of RA; they can efficiently attenuate disease symptoms and substantially decrease and/or delay joint deformity [4]. DMARDs can be classified as follows: conventional DMARDs and biologic DMARDs [5]. Commonly used conventional DMARDs include methotrexate, leflunomide, hydroxychloroquine, and sulfasalazine. Recently, many biological DMARDs, including TNF inhibitors (adalimumab, infliximab, and etanercept), anti-CD20 antibodies (rituximab), IL-6 receptor antibodies (sarilumab), RANKL antibodies (denosumab), and Janus kinase inhibitors (baricitinib), have been developed [6, 7]. Despite the increasing numbers of new drugs and treatment regimens, agents that completely cure RA or long-acting agents for RA are still far from being developed; thus, novel therapeutics and/or targets for this condition are required.

Hereditary factors show a clear causal relationship with RA [8]. And elucidating the pathogenesis of RA from the genomics and genetics standpoints is an important means for clinical therapeutics and drug discovery [9]. At present, genome-wide association studies (GWASs) have identified over 100 genetic loci associated with RA susceptibility [10, 11]. Although genetic information indicates an association between genetic factors and RA, the most causal risk genes (RGs) associated with RA and the molecular mechanisms underlying this disease remain unknown [12]. Mo et al. [13] predicted the RA-associated susceptibility genes by the summary data-based Mendelian randomization (SMR) analysis and identified 140 genes that showed causal association with RA. Moreover, thus far, only a few effective drug targets have been identified through GWASs [14].

**Figure 1:**
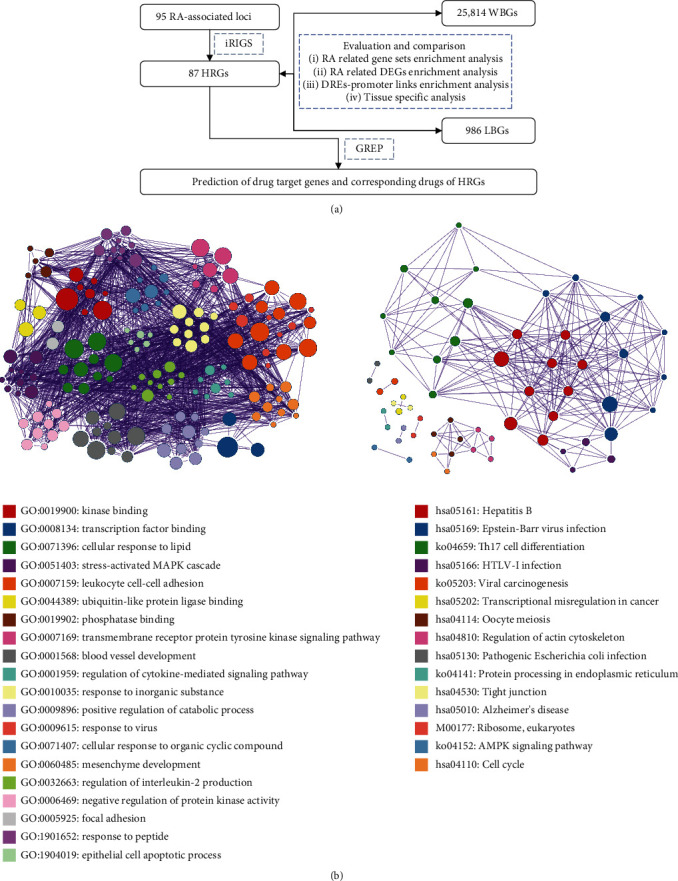
A flowchart depicting the steps in our study and the function enrichment analysis of the HRGs. (a) A flowchart detailing the steps followed in this study. (b) The GO and KEGG pathway analyses of the HRGs.

In this study, to identify RA-associated RGs and predict candidate drug targets for RA, we collected 95 RA-associated loci from different GWASs and detected the candidate RGs from these loci via integrated multiomics data (the genome-scale chromosome conformation capture data, enhancer-promoter linkage data, and gene expression data) using the Bayesian integrative risk gene selector (iRIGS) [15]. Then, we evaluated the relevance between the candidate RGs and RA progression in the context of multiple aspects, such as biological functions, gene expression, and gene regulatory patterns. Finally, we predicted the candidate targets and drugs of these RA-associated RGs using the drug repositioning prediction approach (Figure 1(a)).

## 2. Methods

### 2.1. RA-Associated Loci

We collected over 100 RA-associated loci from multiple GWASs, including 101 loci collected from a meta-analysis GWAS containing over 100,000 subjects of European and Asian ancestries (29,880 RA cases vs. 73,758 controls) [16], two loci collected from a GWAS containing over 1,600 subjects (397 RA cases vs. 1,211 controls) [17], and four loci collected from a case-control GWAS of a cohort of Arab subjects (511 RA cases vs. 352 controls) [18]. Finally, a total of 104 RA-associated loci were collected (there are 3 duplicated SNPs). After excluding 12 loci for which SNP IDs were unavailable, 95 RA-associated loci were included in this study.

### 2.2. Identifying RGs for the RA-Associated Loci

The high-confidence risk genes (HRGs) of RA were inferred by iRIGS (GRCh38/hg38) [15], which is a powerful tool for RG identification that integrates multiomics data and gene networks. Here, the omics data include two RA-associated gene expression datasets, i.e., GSE55235 [19] and GSE77298 [20], two distal regulatory element- (DRE-) promoter linkage datasets, 1,618,000 DRE promoter linkages obtained from genome-scale chromosome conformation capture (Hi-C) [21], and 66,899 enhancer-promoter linkages obtained from the FANTOM5 project [22]. All these omics data have been processed and deposited in iRIGS. Furthermore, the GO network data containing gene-gene relationships obtained by the iRIGS method were also integrated. A total of 1,972 candidate genes located within a 2 Mb region centered at the index SNP were collected as the candidate genes for iRIGS analysis. The posterior probability (PP) value was calculated by a Bayesian framework embedded in iRIGS [15], which is the index of possibility for genes to serve as an RG for RA. For each GWAS locus, one or more RGs can be selected according to the *PP* value. In this study, we only selected one risk gene with the highest *PP* for each locus. For evaluation of HRGs, we constructed two background gene lists for comparison with the HRGs: (1) the local background genes (LBGs), which is defined as the genes with *PP* values less than the median *PP* of all candidate genes (1,972 genes located within a 2 Mb region of the RA-associated loci). Ultimately, a total of 986 LBGs were obtained; (2) the whole-genome background genes (WBGs), which are defined as the genes that included all the human genes (obtained from the R package of iRIGS) except the HRGs. Ultimately, a total of 25,814 WBGs were obtained.

### 2.3. Data Collection

Five RA-associated keyword gene sets (keywords: “Arthritis,” “Rheumatic,” “Autoimmune,” “Joint,” and “Connective Tissue”) were constructed from the GeneCards database (http://www.genecards.org). At first, the five keywords were used to research the related genes in the GeneCards database; then, the genes with a relevance score greater than 10 were considered as the keyword-related genes. Finally, it was found that the “Connective Tissue” gene set contained 507 genes, the “Joint” gene set contained 1,063 genes, the “Autoimmune” gene set contained 457 genes, the “Arthritis” gene set contained 422 genes, and the “Rheumatic” gene set contained 65 genes. Furthermore, an immune system-related gene set containing 1,534 genes was collected from the ImmPort database (https://www.immport.org) [23]. The tissue-specific gene expression profiles (FPKM, reads per kilobase of transcript per million mapped reads) were collected from GTEx release V8 data source [24].

### 2.4. Drug Repositioning Prediction of the HRGs

To predict the drug-specific target genes and corresponding drugs specific to the HRGs, a command-line Python software, Genome for REPositioning drugs (GREP), was used [25]. The GREP software quantifies the enrichment of drug targets by using DrugBank and the Therapeutic Target Database. Approximately 22,300 drugs and 2,029 genes were categorized based on the Anatomical Therapeutic Chemical (ATC) and World Health Organization (WHO) classification system; the *P* values and odds ratios for this categorization were calculated using Fisher's exact test.

### 2.5. Statistical Analysis

The differentially expressed genes (DEGs) were identified using the Limma package in the R software (adjusted. *P* < 0.05) [26]. The GO and pathway enrichment analyses were performed using Metascape [27]. One-sided Fisher's exact test and one-sided Wilcoxon rank-sum test were performed using the R software. The Jensen–Shannon divergence (JSD) score was calculated using the R package “philentropy.” The *P* values were adjusted using the Bonferroni correction method.

## 3. Results

### 3.1. Predicting HRGs for RA

A total of 87 HRGs related to the 95 RA-associated loci were inferred using iRIGS; most of these genes have been implicated in RA and/or autoimmunity (see Table 1 and Supplementary Table 1). Some of the well-known drug targets for RA treatment, such as IRAK1, HIF1A, and IL6ST, have been identified as HRGs for RA [28]. Further, 36 and 46 genes have been reported to be related to RA and autoimmunity, respectively. For instance, IL6/IL6ST signaling plays a key role in the progression of RA, and some IL6 receptor antagonists have been proved to be effective in altering leukocyte trafficking and reducing the severity of RA [29]. GATA-3 has been shown to protect against severe joint inflammation and reduce the differentiation of Th17 cells in mice with RA [30]. EGR2 acts as a key regulator for systemic autoimmunity by regulating cytokine production and cell proliferation [31]. Meanwhile, we also investigated the rest HRGs which have no direct evidence linking to RA and found that these HRGs might also be close to RA or autoimmunity diseases (Table 2). For example, PTPRC is associated with response to antitumor necrosis factor-alpha therapy, which is a mainstay of treatment in rheumatoid arthritis [32]. ANXA11 is an antigen associated with multiple systemic autoimmune diseases [33]. GDI2 is a candidate biomarker in synovial fluid of RA [34]. And there are seven genes (TNFAIP3, XPO1, GDI2, GATA3, EGR2, DDB1, and ABI2) supported by more than one SNP. Most of which are related to the RA. TNFAIP3 showed differential expression between RA and osteoarthritis synoviocytes [35]. XPO1 has been indicated to serve as new candidate therapeutic targets for RA [36]. Moreover, the GO and KEGG pathway enrichment analyses of the HRGs showed that these genes were enriched mainly in intercellular communication and immune-related functions and pathways, such as leukocyte cell-cell adhesion, focal adhesion, regulation of cytokine-mediated signaling pathways, tight junction formation, Th17 cell differentiation, and regulation of interleukin-2 production (Figure 1(b)). These functions and pathways have been reported to be critical for RA progression [37, 38].

### 3.2. Evaluation of the HRGs

**Figure 2:**
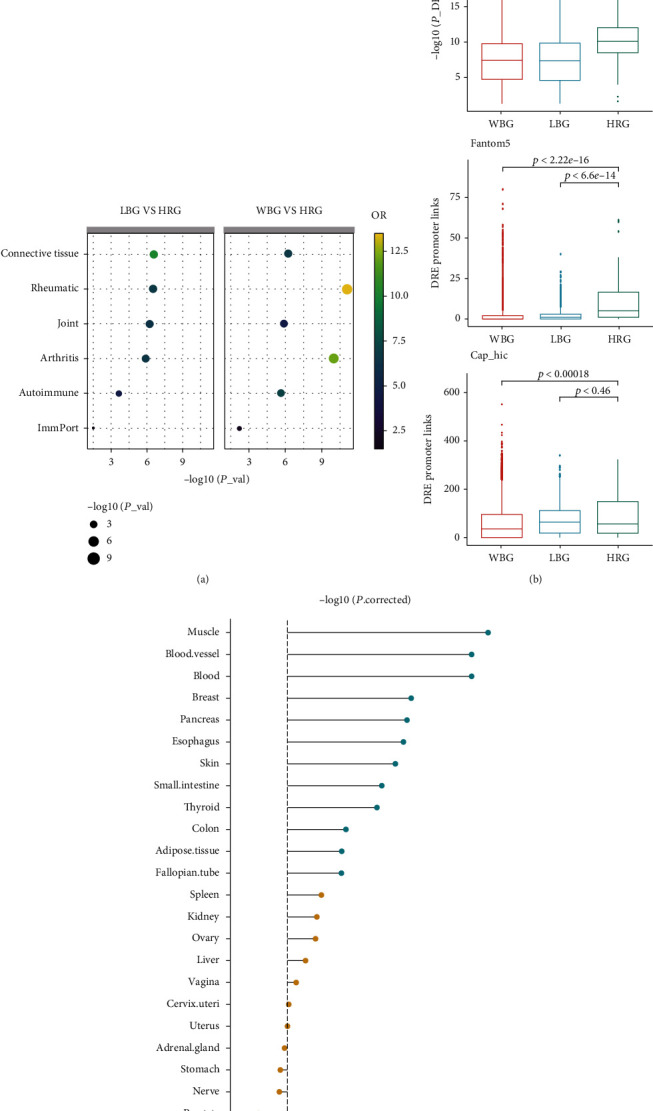
Comparison of the HRGs with the local background genes (LBGs) and whole-genome background genes (WBGs). (a) Comparison of the HRGs with the LBGs and WBGs using the six RA-related gene sets: the “Arthritis,” “Rheumatic,” “Autoimmune,” “Joint,” “Connective Tissue,” and “ImmPort” gene sets. (b) Comparison of the HRGs with the LBGs and WBGs using the two gene expression datasets GSE77298 and GSE55235 and the two DRE-promoter linkage datasets obtained using the Hi-C and FANTOM5. (c) Tissue-specificity analysis of the HRGs (one-sided Wilcoxon rank-sum test).

To assess the reliability of the HRGs, we constructed two background gene lists for comparison with the HRGs: the local background genes (LBGs) included 986 genes with *PP* values less than the median *PP* of all candidate genes, and the whole-genome background genes (WBGs) included all the human genes except the HRGs (25,814 genes). At first, concerning biological function, we compared the HRGs with the LBGs and WBGs using the six RA-related gene sets, i.e., the “Arthritis,” “Rheumatic,” “Autoimmune,” “Joint,” “Connective Tissue,” and “ImmPort” gene sets (see Methods for details). As shown in Figure 2(a), HRGs were significantly enriched in all the six RA-related gene sets (one-sided Fisher's exact test: *P* value < 0.05). Next, about gene expression, we compared the HRGs with the LBGs and WBGs using the two gene expression datasets GSE77298 and GSE55235; as shown in Figure 2(b), the HRGs were more likely to serve as the DEGs in these two RA gene expression profiles (one-sided Wilcoxon rank-sum test: *P* value < 0.05). Then, with regard to gene regulation, we compared the HRGs with the LBGs and WBGs using the two DRE-promoter linkage datasets obtained using the Hi-C and FANTOM5 methods. These results also showed that the HRGs were significantly associated with a large number of DREs (Figure 2(b); one-sided Wilcoxon rank-sum test: *P* value < 0.05). To investigate the tissue specificity of the HRGs, we converted the RPKM GTEx data to JSD scores to represent the tissue specificity of each gene for each tissue. Moreover, compared to the LBGs, the HRGs showed a significantly high expression in the muscles, blood vessels, blood, etc. (see Figure 2(c), one-sided Wilcoxon rank-sum test: adjusted *P* value < 0.05). These tissues have been proved involved in RA progression. For example, muscle deterioration (myositis and weakness) and inflammation of blood vessels (vasculitis and ulcers) are common complications of RA [39].

### 3.3. Predicting the Targets and Corresponding Drugs for the HRGs

**Figure 3:**
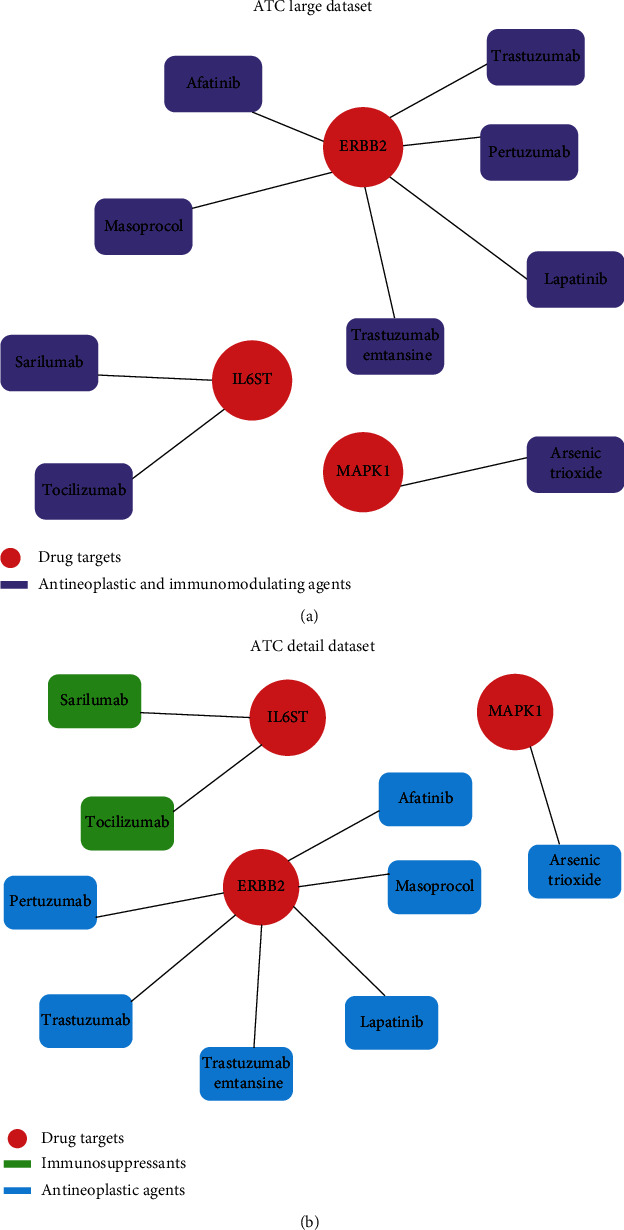
Drug repositioning prediction of the HRGs based on (a) the ATC large dataset and (b) the detailed ATC dataset.

To investigate whether some HRGs could serve as targets of existing repositioned drugs for RA therapy, we used GREP to perform enrichment analysis to ascertain the targets of the existing and approved drugs (see Methods for details). As shown in Figure 3 and Supplementary Table 2, three HRGs, ERBB2, IL6ST, and MAPK1, were identified to be related to the targets of immunosuppressants and antineoplastic agents. A total of six potential drugs (trastuzumab, pertuzumab, trastuzumab emtansine, lapatinib, afatinib, and masoprocol) were predicted to target ERBB2. Of these, trastuzumab, pertuzumab, and trastuzumab emtansine are HER2/ErbB2 receptor monoclonal antibodies approved for the treatment of metastatic HER2-positive breast cancer, and trastuzumab has been reported to inhibit RA synovial cell growth [40]. Lapatinib has been reported to ameliorate experimental arthritis in rats by targeting epidermal growth factor receptors (EGFRs) [41]. Li et al. [42] found that masoprocol significantly reduces the severity of bone destruction and osteoclast recruitment in the ankle joint of rats with adjuvant-induced arthritis and indicated the potential utility of masoprocol as a therapeutic agent for RA. Pertuzumab and afatinib have also been approved as antineoplastic agents. Two potential drugs (tocilizumab and sarilumab) were predicted to target IL6ST. Tocilizumab, which functions by targeting IL-6 receptors, was the first DMARD to be approved for RA treatment [43]. Sarilumab was the second IL-6 receptor antagonist to be approved for the treatment of RA [44]. Arsenic trioxide, which has been reported as a potential therapeutic agent for RA, was predicted to target MAPK1; it has also been approved to treat leukemia and reported to regulate the Treg and Th17 cell balance by modulating STAT3 expression in treatment-naïve RA patients [45].

## 4. Discussion

To date, the exact cause of the immune system's faulty response in RA remains unclear [46]. Though some genes have been identified to be responsible for the increased risk of developing RA, such as HLA complex, STAT4, TRAF1, and PTPN22 [47], most RA-related RGs and their causal variants remain unknown [48]. Recently, GWASs have been utilized to identify RA-associated genetic variants on a genome-wide scale, and over 100 RA-associated loci were obtained [10, 11]. However, the presence of most GWAS variants (90%) in noncoding regions hinders the identification of disease-related RGs [49], which also obscures the interpretation of their mode of action and the correct identification of the target gene via which the causal variant may affect the phenotype [50]. Herein, to fill this gap, we identified 87 HRGs from 95 RA-associated loci collected from different GWASs based on multiomics data. The assessment of the HRGs indicated that they were markedly correlated with RA progression. In addition, using drug repositioning prediction, we also identified several targets of these genes and the drugs associated with their function. Some of these identified drugs have already been approved for RA treatment.

The inspection of previously published literature revealed that 36 and 46 HRGs have been implicated in RA progression and autoimmunity, respectively. Besides the well-known drug targets for RA treatment, such as IRAK1, HIF1A, and IL6ST, some HRGs, including XPO1, GATA3, MYC, and CD40, have also been indicated to serve as new candidate therapeutic targets for RA [36, 51, 52]. The function enrichment analysis of the HRGs showed that they were enriched mainly in the immune system- and intercellular communication-related functions and pathways. It is known that RA is a classic autoimmune and inflammatory disease that strongly involves multiple innate and adaptive immune-related processes [53]. Additionally, the dysfunction of several intercellular signaling pathways, including the JAK/STAT, SAPK/MAPK, and PI-3K/AKT/mTOR signaling pathways, plays a critical role in RA [37]. Cell-cell crosstalk mediates various biological processes in the tissue microenvironment in RA. Therefore, many studies have focused on the development of new therapeutics for RA by considering the intercellular communications in RA [54–56]. These results indicate that the HRGs identified herein are markedly involved in RA progression and are of importance for research regarding the mechanism underlying RA and therapeutic strategies for this condition. Moreover, some of the rest HRGs without direct evidence linking to RA are also involved in autoimmunity disease-related functions or pathways. This part of HRGs is probably more worth exploring than the well-known RA-related HRGs.

The comparison of the HRGs with the LBGs and HRGs showed that the HRGs are markedly associated with RA-related functions and RA-related DEGs and indicated that the expression levels of the HRGs tend to be regulated by DREs. Interestingly, the HRGs showed a markedly high expression in the muscle tissues, blood vessels, and blood. Muscle deterioration (myositis and weakness) and inflammation of blood vessels (vasculitis and ulcers) are common complications of RA [39]. Therefore, the high expression of HRGs in these tissues may implicate them in the progression of RA and may highlight them as potential therapeutic targets for RA. Further, the expression of HRGs in the blood may mainly influence RA-related immune processes [57, 58]; this may also implicate these HRGs as factors governing, and ultimately, as candidate biomarkers for, the progression of RA.

Drug repositioning prediction of the HRGs yielded three targets and nine drugs. Two IL-6 receptor antagonist drugs, tocilizumab and sarilumab, have been approved for RA treatment. Meanwhile, trastuzumab, lapatinib, masoprocol, and arsenic trioxide have been reported to ameliorate the symptoms of RA in patients or model animals and may serve as candidate DMARDs for RA treatment. The other drugs, pertuzumab, trastuzumab emtansine, and afatinib, have also been approved as immunosuppressants and/or antineoplastic agents. These results not only indicate that these HRGs are markedly involved in RA progression but also provide a trajectory for screening effective drugs for RA treatment.

## 5. Conclusion

In this study, we collected 95 RA-associated loci from different GWASs of RA and obtained 87 HRGs from these loci using a multiomics-based method. The analysis and evaluation of these HRGs indicated that these genes were indeed, highly involved in RA. Moreover, the drug repositioning prediction of the HRGs suggested several potential targets and drugs for RA treatment. In summary, this study predicted new RGs, drug targets, and drugs for RA using the GWAS and multiomics data. We believe that our study provides more clues for understanding the pathogenesis of RA and will be important for research regarding the mechanisms underlying RA and the possible therapeutic strategies for this condition.

## Figures and Tables

**Table 1:** Information of some RA or autoimmunity-related HRGs.

HRG	SNP	PMID	RA related	Autoimmunity related
IL6ST	rs7731626	16646038	Yes	Yes
SUMO1	rs6715284	30562482; 17360386	Yes	
XPO1	rs13385025, rs34695944	24965445	Yes	
FOXO1	rs9603616	24812285	Yes	Yes
HIF1A	rs3783782	27445820	Yes	Yes
DUSP22	rs9378815	29287311	Yes	
GATA3	rs12413578, rs3824660	19248112; 29097726	Yes	Yes
AKT1	rs2582532	28559961	Yes	
CD40	rs4239702	28455435	Yes	Yes
EGR2	rs6479800, rs71508903	24058814		Yes

**Table 2:** Information of some HRGs without direct evidence linking to RA.

HRGs	SNP	*PP* value	Description
PTPRC	rs17668708	0.429	Associated with response to TNF*α* therapy
ANXA11	rs726288	0.427	Antigen associated with systemic autoimmune diseases
SPRED1	rs8032939	0.369	Suppressor of the Ras–ERK pathway
PRDM1	rs9372120	0.366	PRDM1 is belonging to the B cell development pathway
BUB1	rs6732565	0.351	Differentially expressed in RA chondrocytes
LCLAT1	rs10175798	0.327	Related to triacylglycerol biosynthesis and fatty acyl-CoA biosynthesis
AZI2	rs3806624	0.292	Activator of NFKB
GDI2	rs947474	0.284	Is a candidate biomarker in synovial fluid of RA
CNOT6L	rs10028001	0.2766	Differentially expressed in RA
RFTN1	rs4452313	0.271	Involved in T-cell antigen receptor-mediated signaling

## Data Availability

The data used to support the findings of this study are included within the article.

## Supplementary Materials

Supplementary Materials Supplementary Table 1: list of the 87 HRGs. Supplementary Table 2: predicting the targets and corresponding drugs for the HRGs by GREP.
