# Supplementary material for: Prediction of New Risk Genes and Potential Drugs for Rheumatoid Arthritis from Multiomics Data
Source: Comput Math Methods Med. 2022 Jan 31;2022:6783659. doi: 10.1155/2022/6783659 (PMC8820924; doi:10.1155/2022/6783659)
Supplement: Supplementary Materials — Supplementary Table 1: list of the 87 HRGs. Supplementary Table 2: predicting the targets and corresponding drugs for the HRGs by GREP. [file 6783659.f1.docx]

**Tables**

| Supplementary Table 1. LIST OF THE 87 HRGs. | | | | | |
| --- | --- | --- | --- | --- | --- |
| HRGs | post_prob | SNP | PMID | RA related | Autoimmunity related |
| ARRB2 | 0.067773167 | rs72634030 | 21855149 | Y | Y |
| ZFP36L1 | 0.177389532 | rs1950897 | 30622281 | Y | Y |
| LITAF | 0.204702628 | rs4780401 | 22160695 | Y | Y |
| TRAF6 | 0.670816044 | rs331463 | 25522907 | Y | Y |
| IL6ST | 0.1850768 | rs7731626 | 16646038 | Y | Y |
| ETS1 | 0.467496542 | rs73013527 | 11229456 | Y | Y |
| ERBB2 | 0.092669433 | rs1877030 | 16134726 | Y | Y |
| SUMO1 | 0.146611342 | rs6715284 | 30562482；17360386 | Y | Y |
| STAT6 | 0.094052559 | rs1633360 | 27942004 | Y | Y |
| SOCS6 | 0.375700662 | rs2469434 | 24595859 | Y | Y |
| ADAR | 0.077455048 | rs2228145 | 26500897 | Y | Y |
| RPS25 | 0.092669433 | rs10790268 | 32945465 | Y | Y |
| IRAK1 | 0.070539419 | rs5987194 | 18759964 | Y | Y |
| RBPJ | 0.491155274 | rs11933540 | 26604133 | Y | Y |
| MYC | 1 | rs1516971 | 15457447 | Y | Y |
| RAB14 | 0.157676349 | rs10985070 | 16254600 | Y | Y |
| XPO1 | 0.294387421 | rs13385025 | 24965445 | Y | Y |
| XPO1 | 0.244813278 | rs34695944 | 24965445 | Y | Y |
| MAPK8 | 0.257261411 | rs2671692 | 18187523 | Y | Y |
| FOXO1 | 0.349930844 | rs9603616 | 24812285 | Y | Y |
| TNFAIP3 | 0.421853389 | rs10499194 | 20822710；32664585；26405544 | Y | Y |
| TNFAIP3 | 0.369294606 | rs17264332 | 20822710；32664585；26405544 | Y | Y |
| TNFAIP3 | 0.365145228 | rs6920220 | 20822710；32664585；26405544 | Y | Y |
| TNFAIP3 | 0.435932154 | rs7752903 | 20822710；32664585；26405544 | Y | Y |
| HIF1A | 0.421169105 | rs3783782 | 27445820 | Y | Y |
| DUSP22 | 0.30428769 | rs9378815 | 29287311 | Y | Y |
| HOXA11 | 0.114799447 | rs67250450 | 26605300 | Y | Y |
| STAT1 | 0.307053942 | rs11889341 | 14962955 | Y | Y |
| GATA3 | 0.733056708 | rs12413578 | 19248112; 29097726 | Y | Y |
| GATA3 | 0.412899469 | rs3824660 | 19248112; 29097726 | Y | Y |
| PPP1CC | 0.156249545 | rs10774624 | 23396208 | Y | Y |
| KRIT1 | 0.138312586 | rs4272 | 22922958 | Y | Y |
| CTSB | 0.263580112 | rs2736337 | 25889265 | Y | Y |
| AKT1 | 0.254495159 | rs2582532 | PMC3272755 | Y | Y |
| YWHAZ | 0.339288054 | rs678347 | 32407294 | Y | Y |
| WDR1 | 0.394190871 | rs13142500 | 26008898 | Y | Y |
| CD40 | 0.096818811 | rs4239702 | 28455435 | Y | Y |
| RUNX1 | 0.345606755 | rs8133843 | 15225361 | Y | Y |
| ABCG1 | 0.152478707 | rs1893592 | 29905812 | Y | Y |
| NCSTN | 0.051175657 | rs4656942 | 33911744 | Y | Y |
| MAPK1 | 0.282157676 | rs11089637 | 16424221 | Y | Y |
| EGR2 | 0.300138313 | rs6479800 | 24058814 |  | Y |
| EGR2 | 0.386896702 | rs71508903 | 24058814 |  | Y |
| MACF1 | 0.110650069 | rs12140275 | 29373494 |  | Y |
| RPS6KA2 | 0.362378976 | rs1571878 | 29310926 |  | Y |
| MAPK14 | 0.163208852 | rs2234067 | 25502009 |  | Y |
| ADARB1 | 0.107447041 | rs2236668 | 23001123 |  | Y |
| PARK7 | 0.255426949 | rs227163 | 26634899 |  | Y |
| ANXA5 | 0.169323724 | rs45475795 | 32111835 |  | Y |
| DDX17 | 0.069156293 | rs909685 | 15373920 |  | Y |
| NCK1 | 0.416320885 | rs9826828 | 32427580 |  | Y |
| HEY1 | 0.326417704 | rs998731 | 30279738 |  | Y |
| CNOT6L | 0.276625173 | rs10028001 |  |  |  |
| LCLAT1 | 0.327786271 | rs10175798 |  |  |  |
| VCP | 0.154910097 | rs11574914 |  |  |  |
| ARAP1 | 0.124364854 | rs11605042 |  |  |  |
| COX4I1 | 0.211618257 | rs13330176 |  |  |  |
| PTPRC | 0.429060202 | rs17668708 |  |  |  |
| RAB1A | 0.26417704 | rs1858037 |  |  |  |
| ABI2 | 0.24757953 | rs1980422 |  |  |  |
| KLHL20 | 0.179806362 | rs2105325 |  |  |  |
| CDC5L | 0.105117566 | rs2233424 |  |  |  |
| EIF1AX | 0.162291621 | rs2301888 |  |  |  |
| HDGF | 0.123098202 | rs2317230 |  |  |  |
| EZR | 0.163208852 | rs2451258 |  |  |  |
| HIPK1 | 0.136929461 | rs2476601 |  |  |  |
| PAM | 0.393346437 | rs2561477 |  |  |  |
| ATP10D | 0.260027663 | rs2664035 |  |  |  |
| INPP5B | 0.141078838 | rs28411352 |  |  |  |
| ABI2 | 0.232365145 | rs3087243 |  |  |  |
| RAC2 | 0.076071923 | rs3218251 |  |  |  |
| SMARCA4 | 0.084370678 | rs34536443 |  |  |  |
| AZI2 | 0.291839557 | rs3806624 |  |  |  |
| CEP57 | 0.244201791 | rs4409785 |  |  |  |
| RFTN1 | 0.271689597 | rs4452313 |  |  |  |
| DDB1 | 0.131396957 | rs508970 |  |  |  |
| MAN1A2 | 0.195020747 | rs624988 |  |  |  |
| HINT1 | 0.109266943 | rs657075 |  |  |  |
| BUB1 | 0.350877193 | rs6732565 |  |  |  |
| GDI2 | 0.225449516 | rs706778 |  |  |  |
| ANXA11 | 0.427385892 | rs726288 |  |  |  |
| ATF6 | 0.065006916 | rs72717009 |  |  |  |
| ARF4 | 0.230982019 | rs73081554 |  |  |  |
| SON | 0.147994467 | rs73194058 |  |  |  |
| STAT2 | 0.057814661 | rs773125 |  |  |  |
| MTPAP | 0.328033777 | rs793108 |  |  |  |
| TLE3 | 0.2611924 | rs8026898 |  |  |  |
| SPRED1 | 0.369294606 | rs8032939 |  |  |  |
| RNMT | 0.147994467 | rs8083786 |  |  |  |
| RPS18 | 0.059474412 | rs9268839 |  |  |  |
| PRDM1 | 0.366528354 | rs9372120 |  |  |  |
| TAB2 | 0.197786999 | rs9373594 |  |  |  |
| GDI2 | 0.284836573 | rs947474 |  |  |  |
| RPL31 | 0.243371915 | rs9653442 |  |  |  |
| DDB1 | 0.085753804 | rs968567 |  |  |  |

| Supplementary Table 2. Predicting the targets and corresponding drugs for the HRGs by GREP (based on ATC detail dataset). | | | | |
| --- | --- | --- | --- | --- |
| #Group | GroupName | OddsRatio | FisherExactP | TargetGene:DrugNames |
| L04 | IMMUNOSUPPRESSANTS | 2.132609 | 0.39021 | IL6ST:tocilizumab,sarilumab |
| L01 | ANTINEOPLASTIC AGENTS | 1.342466 | 0.460237 | ERBB2:trastuzumab,pertuzumab,trastuzumab emtansine,lapatinib,afatinib,masoprocol;MAPK1:arsenic trioxide |
| A01 | STOMATOLOGICAL PREPARATIONS | 0 | 1 |  |
| A02 | DRUGS FOR ACID RELATED DISORDERS | 0 | 1 |  |
| A03 | DRUGS FOR FUNCTIONAL GASTROINTESTINAL DISORDERS | 0 | 1 |  |
| A04 | ANTIEMETICS AND ANTINAUSEANTS | 0 | 1 |  |
| A05 | BILE AND LIVER THERAPY | 0 | 1 |  |
| A06 | DRUGS FOR CONSTIPATION | 0 | 1 |  |
| A07 | ANTIDIARRHEALS, INTESTINAL ANTIINFLAMMATORY/ANTIINFECTIVE AGENTS | 0 | 1 |  |
| A08 | ANTIOBESITY PREPARATIONS, EXCL. DIET PRODUCTS | 0 | 1 |  |
| A09 | DIGESTIVES, INCL. ENZYMES | 0 | 1 |  |
| A10 | DRUGS USED IN DIABETES | 0 | 1 |  |
| A11 | VITAMINS | 0 | 1 |  |
| A12 | MINERAL SUPPLEMENTS | 0 | 1 |  |
| A14 | ANABOLIC AGENTS FOR SYSTEMIC USE | 0 | 1 |  |
| A16 | OTHER ALIMENTARY TRACT AND METABOLISM PRODUCTS | 0 | 1 |  |
| B01 | ANTITHROMBOTIC AGENTS | 0 | 1 |  |
| B02 | ANTIHEMORRHAGICS | 0 | 1 |  |
| B03 | ANTIANEMIC PREPARATIONS | 0 | 1 |  |
| B05 | BLOOD SUBSTITUTES AND PERFUSION SOLUTIONS | 0 | 1 |  |
| B06 | OTHER HEMATOLOGICAL AGENTS | 0 | 1 |  |
| C01 | CARDIAC THERAPY | 0 | 1 |  |
| C02 | ANTIHYPERTENSIVES | 0 | 1 |  |
| C03 | DIURETICS | 0 | 1 |  |
| C04 | PERIPHERAL VASODILATORS | 0 | 1 |  |
| C05 | VASOPROTECTIVES | 0 | 1 |  |
| C07 | BETA BLOCKING AGENTS | 0 | 1 |  |
| C08 | CALCIUM CHANNEL BLOCKERS | 0 | 1 |  |
| C09 | AGENTS ACTING ON THE RENIN-ANGIOTENSIN SYSTEM | 0 | 1 |  |
| C10 | LIPID MODIFYING AGENTS | 0 | 1 |  |
| D01 | ANTIFUNGALS FOR DERMATOLOGICAL USE | 0 | 1 |  |
| D02 | EMOLLIENTS AND PROTECTIVES | 0 | 1 |  |
| D03 | PREPARATIONS FOR TREATMENT OF WOUNDS AND ULCERS | 0 | 1 |  |
| D04 | ANTIPRURITICS, INCL. ANTIHISTAMINES, ANESTHETICS, ETC. | 0 | 1 |  |
| D05 | ANTIPSORIATICS | 0 | 1 |  |
| D06 | ANTIBIOTICS AND CHEMOTHERAPEUTICS FOR DERMATOLOGICAL USE | 0 | 1 |  |
| D07 | CORTICOSTEROIDS, DERMATOLOGICAL PREPARATIONS | 0 | 1 |  |
| D08 | ANTISEPTICS AND DISINFECTANTS | 0 | 1 |  |
| D09 | MEDICATED DRESSINGS | 0 | 1 |  |
| D10 | ANTI-ACNE PREPARATIONS | 0 | 1 |  |
| D11 | OTHER DERMATOLOGICAL PREPARATIONS | 0 | 1 |  |
| G01 | GYNECOLOGICAL ANTIINFECTIVES AND ANTISEPTICS | 0 | 1 |  |
| G02 | OTHER GYNECOLOGICALS | 0 | 1 |  |
| G03 | SEX HORMONES AND MODULATORS OF THE GENITAL SYSTEM | 0 | 1 |  |
| G04 | UROLOGICALS | 0 | 1 |  |
| H01 | PITUITARY AND HYPOTHALAMIC HORMONES AND ANALOGUES | 0 | 1 |  |
| H02 | CORTICOSTEROIDS FOR SYSTEMIC USE | 0 | 1 |  |
| H03 | THYROID THERAPY | 0 | 1 |  |
| H05 | CALCIUM HOMEOSTASIS | 0 | 1 |  |
| J01 | ANTIBACTERIALS FOR SYSTEMIC USE | 0 | 1 |  |
| J02 | ANTIMYCOTICS FOR SYSTEMIC USE | 0 | 1 |  |
| J04 | ANTIMYCOBACTERIALS | 0 | 1 |  |
| J05 | ANTIVIRALS FOR SYSTEMIC USE | 0 | 1 |  |
| L02 | ENDOCRINE THERAPY | 0 | 1 |  |
| L03 | IMMUNOSTIMULANTS | 0 | 1 |  |
| M01 | ANTIINFLAMMATORY AND ANTIRHEUMATIC PRODUCTS | 0 | 1 |  |
| M02 | TOPICAL PRODUCTS FOR JOINT AND MUSCULAR PAIN | 0 | 1 |  |
| M03 | MUSCLE RELAXANTS | 0 | 1 |  |
| M04 | ANTIGOUT PREPARATIONS | 0 | 1 |  |
| M05 | DRUGS FOR TREATMENT OF BONE DISEASES | 0 | 1 |  |
| M09 | OTHER DRUGS FOR DISORDERS OF THE MUSCULO-SKELETAL SYSTEM | 0 | 1 |  |
| N01 | ANESTHETICS | 0 | 1 |  |
| N02 | ANALGESICS | 0 | 1 |  |
| N03 | ANTIEPILEPTICS | 0 | 1 |  |
| N04 | ANTI-PARKINSON DRUGS | 0 | 1 |  |
| N05 | PSYCHOLEPTICS | 0 | 1 |  |
| N06 | PSYCHOANALEPTICS | 0 | 1 |  |
| N07 | OTHER NERVOUS SYSTEM DRUGS | 0 | 1 |  |
| P01 | ANTIPROTOZOALS | 0 | 1 |  |
| P02 | ANTHELMINTICS | 0 | 1 |  |
| P03 | ECTOPARASITICIDES, INCL. SCABICIDES, INSECTICIDES AND REPELLENTS | 0 | 1 |  |
| R01 | NASAL PREPARATIONS | 0 | 1 |  |
| R02 | THROAT PREPARATIONS | 0 | 1 |  |
| R03 | DRUGS FOR OBSTRUCTIVE AIRWAY DISEASES | 0 | 1 |  |
| R05 | COUGH AND COLD PREPARATIONS | 0 | 1 |  |
| R06 | ANTIHISTAMINES FOR SYSTEMIC USE | 0 | 1 |  |
| R07 | OTHER RESPIRATORY SYSTEM PRODUCTS | 0 | 1 |  |
| S01 | OPHTHALMOLOGICALS | 0 | 1 |  |
| S02 | OTOLOGICALS | 0 | 1 |  |
| S03 | OPHTHALMOLOGICAL AND OTOLOGICAL PREPARATIONS | 0 | 1 |  |
| V03 | ALL OTHER THERAPEUTIC PRODUCTS | 0 | 1 |  |
| V04 | DIAGNOSTIC AGENTS | 0 | 1 |  |
| V06 | GENERAL NUTRIENTS | 0 | 1 |  |
| V08 | CONTRAST MEDIA | 0 | 1 |  |
| V10 | THERAPEUTIC RADIOPHARMACEUTICALS | 0 | 1 |  |
